# Diet drives the gut microbiome composition and assembly processes in winter migratory birds in the Poyang Lake wetland, China

**DOI:** 10.3389/fmicb.2022.973469

**Published:** 2022-09-23

**Authors:** Binhua Wang, Hui Zhong, Yajun Liu, Luzhang Ruan, Zhaoyu Kong, Xiaozhen Mou, Lan Wu

**Affiliations:** ^1^Key Laboratory of Poyang Lake Environment and Resource Utilization, School of Life Science, Ministry of Education, Nanchang University, Nanchang, China; ^2^Department of Biological Sciences, Kent State University, OH, United States

**Keywords:** microbiome, host phylogeny, ecological assembly, network robustness, migratory birds

## Abstract

The complex gut bacterial communities may facilitate the function, distribution, and diversity of birds. For migratory birds, long-distance traveling poses selection pressures on their gut microbiota, ultimately affecting the birds’ health, fitness, ecology, and evolution. However, our understanding of mechanisms that underlie the assembly of the gut microbiome of migratory birds is limited. In this study, the gut microbiota of winter migratory birds in the Poyang Lake wetland was characterized using MiSeq sequencing of 16S rRNA genes. The sampled bird included herbivorous, carnivorous, and omnivorous birds from a total of 17 species of 8 families. Our results showed that the gut microbiota of migratory birds was dominated by four major bacterial phyla: Firmicutes (47.8%), Proteobacteria (18.2%), Fusobacteria (12.6%), and Bacteroidetes (9.1%). Dietary specialization outweighed the phylogeny of birds as an important factor governing the gut microbiome, mainly through regulating the deterministic processes of homogeneous selection and stochastic processes of homogeneous dispersal balance. Moreover, the omnivorous had more bacterial diversity than the herbivorous and carnivorous. Microbial networks for the gut microbiome of the herbivorous and carnivorous were less integrated, i.e., had lower average node degree and greater decreased network stability upon node attack removal than those of the omnivorous birds. Our findings advance the understanding of host-microbiota interactions and the evolution of migratory bird dietary flexibility and diversification.

## Introduction

Migratory birds travel a long distance annually between the breeding grounds, resting areas, and wintering grounds (Kreisinger et al., 2017). This unique behavior exposes migratory birds to complex diets and varying living environments, which may affect the host–microbe interactions and lead to changes in the gut microbial community (McCormick et al., 2013; Grond et al., 2018; Bodawatta et al., 2021). The avian gut microbiome is essential in improving host bird fitness, adaptability, and evolution (Grond et al., 2018). Therefore, understanding factors that impact the assembly of the gut microbiome can help to establish a compressive view of the interactions between migratory birds and their environment. This knowledge will improve our understanding of the health and fitness of migratory birds, particularly in the context of enhanced environmental disturbances and global change.

Both host phylogeny and diet are major regulators of gut microbiota (Hird et al., 2015; Lewis et al., 2016; Zhang et al., 2021). Hosts can serve as a “habitat filter” allowing preferentially colonizing specific microbes in the gut that can provide mutual benefits to the host (John et al., 2010; Stegen et al., 2013; Sieber et al., 2019). Host phylogeny of birds has been found to explain most of the variation in gut microbial composition, followed closely by ecological variables, such as local habitat and diet (Hird et al., 2014; Waite and Taylor, 2014). Several scientists have attempted to link gut microbial community composition to avian phylogenetic identity but yielded no congruent conclusions (Capunitan et al., 2020; Trevellineet et al., 2020). Moreover, a recent study found weak signatures of phylosymbiosis among 15 species of cranes (family Gruidae) housed in the same captive environment and maintained on identical diets (Trevellineet et al., 2020).

Diets are considered one of the main sources of microbial colonizers for guts, as they provide nutrients to both hosts and gut microbes while requiring different digestive requirements assisted by specialized microbial communities (Laparra and Sanz, 2010; Grond et al., 2018). In general, migratory birds are exposed to a wider range of diets (food-associated microorganisms) than nonmigratory birds. Hence, the change in diet due to geographical variation may affect the gut microbiome of migratory birds greater than nonmigratory birds (Grond et al., 2018). We hypothesized that diets play more significant roles than host phylogeny in the gut microbial community of migratory birds. However, which processes drive the assembly and shift of migratory bird gut microbiome remains unknown.

Differential selection of microbes by the host is assumed to drive variation in microbial communities (Mazel et al., 2018). Host hosts may be exposed to the same pool of potential microbial colonizers but then filter and select certain microbes to persist as their symbionts. However, some theoretical studies have suggested that for certain host-associated habitats, such as the vertebrate gut, host selection may not fully explain the observed patterns (Mazel et al., 2018); other ecological and evolutionary processes may also be contributing factors (Kohl, 2020). Stegen et al. (2013) proposed five main mechanisms of microbial community assembly, including variable selection, homogeneous selection, homogeneous dispersal, dispersal limitation, and undominated processes (Stegen et al., 2013). Selection may cause communities to converge if they undergo similar environmental conditions (homogeneous selection) or diverge if they undergo distinct environmental conditions (variable selection; Chase and Myers, 2011; Ge et al., 2021). Depending on the magnitude of dispersal, it can also converge or diverge communities. Ecological drift results in stochastic population fluctuation of component species in communities by chance birth and death events and, thus, generally disperses communities (Ge et al., 2021). Dietary specialization can impact the assembly of the gut microbial community in migratory birds by enhancing the variable selection contribution of specific processes (Kohl, 2020).

We tested the hypothesis using samples from Poyang Lake (PYL; 28°22′-29°45′N, 115°47′-116°45′E) wetland. PYL wetland is one of the world’s six major wetland systems and is located in the lower and middle reaches of the Yangtze River, China (Mei et al., 2016). PYL serves as a critical wintering habitat for migratory birds along the East Asian-Australasian Flyway, which supports about 76 species and 500, 000 individual migratory birds during the wintering period each year (Wang et al., 2016; Yang et al., 2020).

## Materials and methods

### Sample collection

**Figure 1:**
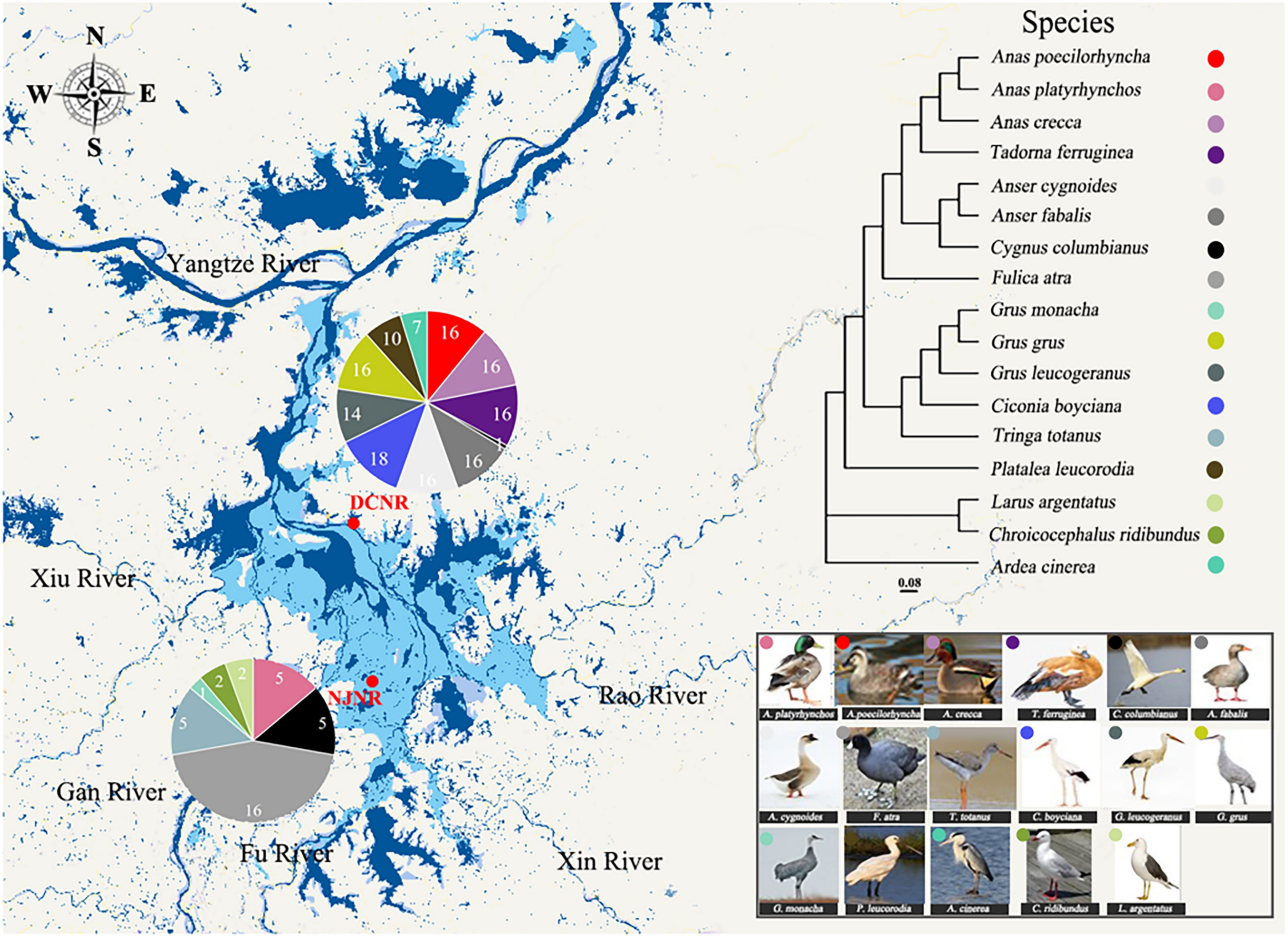
Overview of migratory bird feces samples collected from the Poyang Lake (PYL). Pie charts represent the number of fecal samples from each species at a different site. A phylogenetic tree based on partial mitochondrial gene sequences shows the lineage of the 17 bird species.

This work was performed at the Nanjishan Wetland Nature Reserve (NJNR) and Duchang Migratory bird Nature Reserve (DCNR), which are located in the southwestern and northeastern parts of PYL, respectively (Figure 1). Most of the migratory birds [e.g., Siberian cranes (*Grus leucogetanus*), oriental storks (*Ciconia boyciana*), and swan geese (*Anser cygnoides*)] were distributed in the NJNR and DCNR (Barter et al., 2005; Aharon-Rotman et al., 2017), without significant site differences. These migratory birds typically arrive at PYL starting in October and do not depart until the following March (Xia et al., 2016). There is no geographical isolation between the two sites, and migratory birds can fly freely in between.

Fresh feces of migratory birds were collected from January 2019 to February 2020; this sampling time overlapped with the ongoing NJNR and DCNR monitoring projects. During sampling, locations with large flocks of single bird populations (i.e., > 200 individuals) were chosen for the bird feces collection. All fresh feces were collected and transferred promptly into sterile tubes after the birds had flown away. Specifically, solid feces (excreted by herbivorous and omnivorous birds) were directly picked up from the ground by hands with sterile gloves, while liquid feces (excreted by carnivorous birds) were collected by gently scraping (avoiding obvious soil particles) the soil surface using a sterilized spoon. The freshness of fecal samples was confirmed by observation of instant defecation and the presence of a high moisture sheen (Cox et al., 2005). To ensure samples from multiple individuals, we collected feces at least 2 m away from each other and changed disposable gloves for each sample (Cao et al., 2020). One hundred and eighty samples from 17 species were frozen on dry ice immediately and then stored at –80°C until DNA extraction. Migratory bird species associated with feces samples were identified with the assistance of NJNR and DCNR protection station personnel based on methods described by Barter et al. (2005) and a waterbird survey manual (Bai et al., 2021). Since the samples were collected by picking up the feces of migratory birds, the effects of age and gender on gut microbes were not considered in this study.

### Host phylogeny and classification by diets

The complete mitochondrial 16S rRNA and cytochrome b (Cyt b) gene homologous sequences of migratory bird species were obtained using the National Center for Biotechnology Information (NCBI) blast server.^1^ A phylogenetic tree was created using the Neighbor-Joining algorithm using MEGA X (Kumar et al., 2018).^2^ Sequences of the 16S rRNA region were aligned using MAFFT (Katoh et al., 2002), and the host phylogeny of birds was constructed using Fast Tree (Price et al., 2010). According to their main foraging areas and diets in PYL, the migratory birds were further classified into three guilds: herbivorous, omnivorous, and carnivorous birds (Barzen et al., 2009; Wang et al., 2013; Xia et al., 2016).

### DNA extractions and high-throughput sequencing of fecal microorganisms

DNA was extracted from fresh feces (0.2 g) using the Power Fecal DNA Isolation Kit (MOBIO, United States), following the manufacturer’s instructions (Vo and Jedlicka, 2014). Extracted DNA was quantified using a NanoDrop ND-2000 spectrophotometer (Thermo-Fisher Scientific, California, USA) and then sent to Majorbio Co. Ltd. (Shanghai, China) for 16S rRNA gene (V3-V4 hypervariable region: 338F 5′ACTCCTACGGGAGGCAGCA3′ and 806R 5′ GGACTACHVGGGTWTCTAAT 3′) paired-end sequencing (2 × 300 bp reads) using the Illumina MiSeq platform (Mori et al., 2014). Raw sequences of partial 16S rRNA genes were deposited in the NCBI Sequence Read Archive (SRA) under accession no. PRJNA736842.

### Sequence analysis

The raw data of Illumina sequences were demultiplexed, quality-filtered, and merged using QIIME2 (v2020.02; Bolyen et al., 2019).^3^ The processed sequences were used to reconstruct amplicon sequence variants (ASVs) by the Divisive Amplicon Denoising Algorithm (DADA 2; Callahan et al., 2016). Obtained ASVs were taxonomically annotated by the pre-trained Naive Bayes classifier (Bokulich et al., 2018) based on the database SILVA 138 at a confidence level of 70%. Afterward, non-bacterial sequences such as mitochondrial and chloroplasts were removed according to the annotation results (Glöckner, 2019). The PICRUSt2 (V2.4.1; Douglas et al., 2020) was used to predict the functional potentials of bacterial communities based on 16S rRNA gene sequences, abundance, and the Integrated Microbial Genomes (IMG).^4^

### Statistical analysis

Potential differences in microbial communities among samples were analyzed using nonmetric multidimensional scaling (NMDS) based Bray-Curtis and weighted UniFrac distances using the vegan package in R software (R Core Team, 2016). The alpha-diversity indices, including Shannon, Chao1, Simpson, and Faith’s phylogenetic diversity (Faith’s PD), were calculated based on the ASV level by “adiv,” an R package to analyze biodiversity in ecology (Pavoine, 2021). Furthermore, the phylogenetic generalized linear mixed models were used to test the effect of diet and sampling time on the alpha diversity of the gut microbiome using the R package “lme4” (Douglas et al., 2015). The phylosymbiosis testing was used to examine the potential role of host phylogeny in microbiome structures (Trevellineet et al., 2020; Bodawatta et al., 2021). The values range from-1 (perfect clustering of dissimilar samples) to 1 (perfect clustering of similar samples), with 0 indicating a perfect random association between microbial communities and host phylogeny. The patterns of phylosymbiosis were used to compare the gut microbiota dendrograms with the bird phylogeny *via* the Robinson–Foulds and matching cluster metrics with 100,000 random trees using the Python script (Brooks et al., 2016). To compare the alpha diversity of the multiple diet groups, we did Kruskal–Wallis rank-sum test followed by a pairwise Wilcoxon rank-sum test with the adjustment of *p* values by Benjamini–Hochberg FDR correction at 0.05. A heatmap was generated using R at the phylum level.

The microbial diversity analyses at the ASVs level were performed with sample size and coverage-based integrations of interpolation (rarefaction) and extrapolation (prediction) of the Hill numbers (Hill, 1973; Alberdi and Gilbert, 2019; Roswell et al., 2021) using the R packages “iNEXT” and “iNextPD” (Chao et al., 2014, 2015; Hsieh et al., 2016). Hill numbers were computed using the scaling parameter *q* (Jost, 2007), corresponding to increasing weight on the species abundance and phylogenetic diversity indices. The larger the q value, the greater the importance of ASVs attributed to the abundance of ASVs. When *q* = 0, the rare species (those with low abundance) were counted; when *q* = 1, the common species (those with medium-high abundances) were measured; when *q* = 2, the dominant species (those with very high abundance) were counted.

To predict the potential interactions among individual ASVs, the phylogenetic molecular ecology networks (pMENs) were constructed at the ASV level using random matrix theory (RMT) models after Pearson correlation estimation (Zhou et al., 2011). All molecular ecology networks (MENs) were constructed based on Pearson correlations of the log-transformed ASV abundance, followed by an RMT-based approach that automatically determines the correlation cut-off threshold (Luo et al., 2007; Zhou et al., 2013). To reduce the complexity of the datasets, the ASVs that presented in no more than five samples were removed before the network constructions (Waite et al., 2018). A set of topological features, including average clustering coefficient, average path length, network diameter, graph density, average degree, and modularity, was calculated using “igraph” in the R package (Csardi and Nepusz, 2006). The network plot was visualized with Gephi 0.9.2 software (Bastian et al., 2009). Each node represented one ASV, and each edge represented a strong and significant correlation between two ASVs. Network stability was evaluated by removing nodes in the static network to estimate how quickly robustness degraded, and network robustness was assessed by the nodes’ degree distribution and natural connectivity. The node removal was followed by the random repetitive principle (Peng and Wu, 2016).

### Quantification of ecological processes of microbial communities

Mean-nearest-taxon-distance (MNTD) and the nearest-taxon-index (NTI) were used to characterize the phylogenetic community composition of each sample using “mntd” and “ses. Mntd” in package “Picante” by R (Webb et al., 2002). Nearest-taxon-index quantified the number of standard deviations that the observed MNTD from the mean of the null distribution (999 randomizations). The abundance-weighted mean was then taken across these phylogenetic distances. Null model distribution of βMNTD (Pawlowsky-Glahn and Buccianti, 2011) was built by shuffling bacterial taxa among the tips of the phylogenetic tree or between different communities using randomization and permutation analyses. The β-nearest taxon index(βNTI), a standardized measure of the βMNTD, was then generated by comparing the observed and the null distribution of βMNTD, using the following formula: βNTI = (βMNTDobs-βMNTDnull)/sd (βMNTD_null_).

To examine the potential effects of stochastic and deterministic processes in the assembly of gut microbial communities, we calculated the Levins’ niche breadth (B) index using the “niche width” function in the R package “spaa” (Zhang, 2016). A given ASV with a high B value represents a wide habitat niche breadth. The community-level B value was calculated as the average of B values from all taxa in one given community (Jiao et al., 2019; Mo et al., 2021).

## Results

### Classification of migratory birds

A total of 180 fecal samples were collected. They were from a total of 17 unique bird species of 8 families, including Ardeidae, Anatidae, Ciconiidae, Gruidae, Laridae, Rallidae, Scolopacidae, and Threskiornithidae (Table 1). The branching pattern of the host phylogeny tree illustrated the evolutionary relationships of these species, with the *Anas poecilorhyncha* (APo) and *Anas platyrhynchos* (APl) clustered together as the most closely related but distant from other species (Figure 1). According to the main foraging areas and diets, the populations were divided into three dietary groups: herbivorous (6 species, 69 individuals), omnivorous (5 species, 69 individuals), and carnivorous (6 species, 42 individuals). The herbivorous exploit the stems and leaves of *Carex*, the tubers of *Vallisneria* spp., and *Polygonum criopolitanum*, which are abundant in the PYL wetland. In contrast, carnivores use water invertebrates and fish as their main food. The omnivorous feed on food which the herbivorous and carnivorous also forage.

**Table 1:** Migratory bird species associated information in this study, including general diet, taxonomy, abbreviations, common names, and the number of samples; localities related to samples were mapped in Figure 1.

Diet	Family	Species	Species abbreviations	Common name	Sample size
Herbivorous	*Anatidae*	*Anser fabalis*	AF	Bean goose	16
	*Anatidae*	*Anser cygnoides*	ACy	Swan goose	16
	*Anatidae*	*Cygnus columbianus*	CC	Tundra swan	6
	*Gruidea*	*Grus leucogeranus*	GL	Siberian crane	14
	*Gruidea*	*Grus grus*	GG	Common crane	16
	*Gruidea*	*Grus monacha*	GM	Hooded crane	1
Omnivorous	*Anatidae*	*Anas crecca*	ACr	Common teal	16
	*Anatidae*	*Anas platyrhynchos*	APl	Mallard	5
	*Anatidae*	*Anas poecilorhyncha*	APo	Spot-billed duck	16
	*Anatidae*	*Tadorna ferruginea*	TF	Ruddy shelduck	16
	*Rallidae*	*Fulica atra*	FA	Common coot	16
Carnivorous	*Ardeidae*	*Ardea cinerea*	ACi	Grey heron	7
	*Ciconiidae*	*Ciconia boyciana*	CB	Oriental stork	16
	*Laridae*	*Larus argentatus*	LA	Herring gull	2
	*Laridae*	*Larus ridibundus*	LR	Black-headed gull	2
	*Scolopacidae*	*Tringa totanus*	TT	Common redshank	5
	*Threskiornithidae*	*Platalea leucorodia*	PL	Eurasian spoonbill	10
Total 3	8	17	17	–	180

### Bird gut microbiome diversity

**Figure 2:**
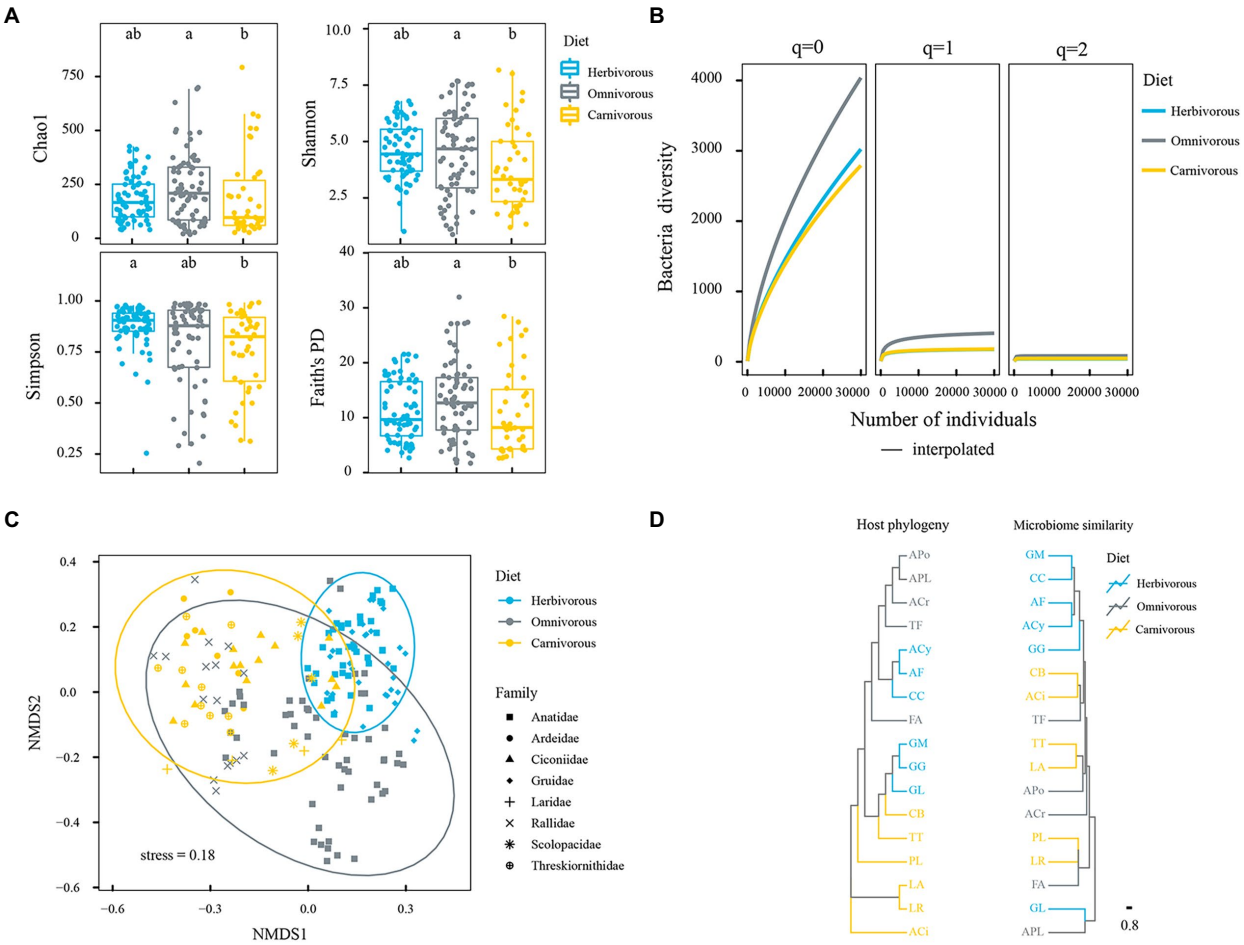
Gut microbial diversity among three diet groups. **(A)** The index of Shannon, Simpson, Chao 1, and Faith’s phylogenetic diversity for each diet group. Results are shown as box and whiskers, the different letters above columns represent the significant difference (*p* < 0.05) by Mann–Whitney *U*-tests adjusted for multiple comparisons; **(B)** coverage based on rarefaction/extrapolation curves of the Hill numbers estimated for three values of the order parameter (*q* = 0, *q* = 1, and *q* = 2). The *x*-axis represents the coverage (that estimates the completeness of the sampling), and the *y*-axis represents the Hill number estimates. 95% confidence interval is also reported; **(C)**: NMDS patterns among diet groups. Ellipses denote a 95% confidence level; **(D)** host phylogeny dendrogram (left panel) obtained using the migratory birds’ partial mitochondrial DNA gene sequences compared to dendrogram (right panel) of weighted UniFrac of gut microbiome at ASVs level. The colours correspond to the trophic categories. Blue represents herbivorous, gray represents omnivorous and yellow represents carnivorous.

A total of 5,460,000 high-quality 16S rRNA gene sequences were recovered, and these were assigned to 6,129 unique amplicon sequence variants (ASVs) after rarefaction. The dilution curve showed an adequate sequencing depth covering most of the diversity of the fecal bacteria community (Supplementary Figure S1). Based on these ASVs, the alpha-diversity indices of the gut microbiome were calculated (Figure 2A, Kruskal–Waillis test, FDR-adjusted *p* = 0.001). Results of phylogenetic generalized linear mixed models revealed that diet had a significant effect on the α-diversity of the gut microbiome (*p* = 0.009). Furthermore, omnivorous species had higher gut microbiome diversity than herbivorous and carnivorous species based on Shannon, Chao1, and Faith’s PD indices. Nonetheless, the α-diversity of gut microbes was significantly different among diet groups, which was mainly caused by rare taxa (Figure 2B, Kruskal–Waillis test, FDR-adjusted *p* = 0.001) as revealed by the value of the order parameter (*q*). Although there was considerable variation among individuals of the same bird population, the species-based difference in α-diversity of gut microbes was still significant. *Larus ridibundus* (LR) had the lowest, but *Anas crecca* (ACr) had the highest gut microbial diversity based on both Shannon and Chao1 indices (Supplementary Table S1; Supplementary Figure S2).

The gut microbial ASVs of the herbivorous were different from those of the carnivorous based on the results of ANOSIM and ADONIS analyses (Supplementary Table S2). The gut microbial community has the greatest difference between herbivorous and carnivorous. Permutational multivariate analysis of variance (PERMANOVA) was performed to determine the relative importance of host phylogeny, diet, sampling time, and their interaction in explaining microbial composition. The results revealed that diet was a major predictor for the gut microbial community of migratory birds (*r*^2^ = 0.201, *p* = 0.001), whereas host phylogeny was a weaker predictor (*r*^2^ = 0.141, *p* = 0.001). The effect of sampling time is small (*r*^2^ = 0.014, *p* = 0.001), which was ignored in the subsequent analysis. The interaction of host phylogeny and diet had a stronger relationship with gut microbial composition than either host species or habitat alone (*r*^2^ = 0.300, *p* = 0.001). In NMDS analysis, the gut microbial assemblage was clustered by diet groups (Figure 2C). Based on the result of topological congruence, the bird phylogeny was not associated with the microbial dendrograms (nRF = 1.0, *p* = 1.0; Figure 2D).

### Bird gut microbial taxonomic composition

**Figure 3:**
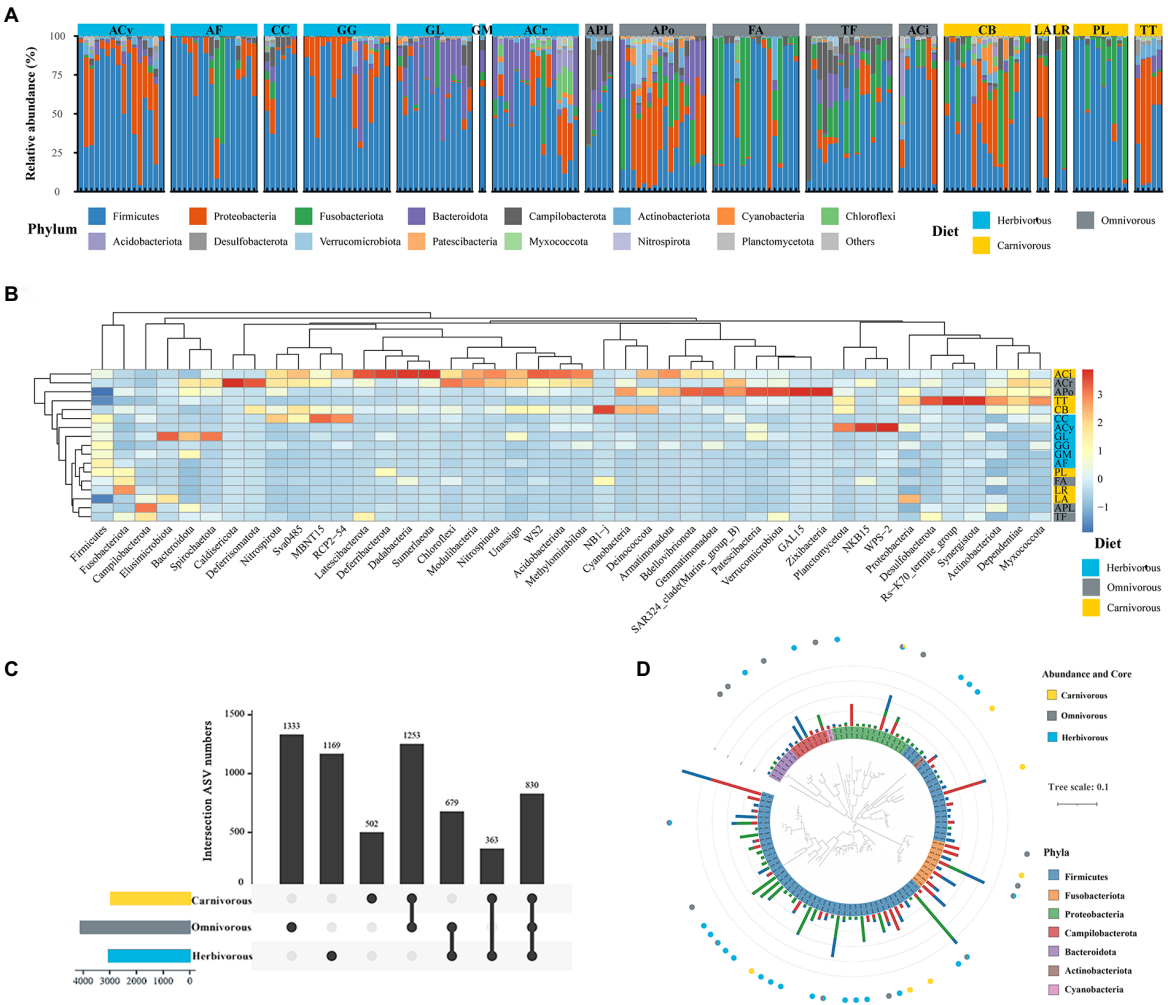
Comparison of gut microbiome among different species. **(A)** Abundance; **(B)** heatmap analysis based on Bray–Curtis distance; **(C)** the number of ASVs that are unique and shared among gut microbiome of three groups; **(D)** Phylogenetic tree of the 100 ASVs with a relative abundance of 0.1% in at least one sample. Bars showed the mean relative abundance of the ASVs. The different diets were labelled with blue, gray and yellow, respectively.

All ASVs of the gut microbiome were restricted within nine bacterial phyla, and four were the most dominant (> 5% of average sequences), including Firmicutes (47.8% of sequences on average), Proteobacteria (18.2%), Fusobacteria (12.6%), and Bacteroidetes (9.1%), accounted for 87.7% of the total sequences collectively (Figure 3A; Supplementary Figure S3). Firmicutes was the most abundant phylum in the gut microbiome in all three diet groups and accounted for 58.0% in the herbivorous, 36.0% in the omnivorous, and 50.0% in the carnivorous. The relative abundance of Fusobacteria in omnivorous (16.5%) and carnivorous (19.8%) birds’ gut microbiome was higher than in the herbivorous (4.4%; Supplementary Figure S2; Krauskal–Waillis test, *p* = 0.001). Furthermore, the results of the heatmap based on Bray–Curtis distance (Figure 3B) revealed the dissimilarity of gut microbial communities among bird species. Firmicutes was the most abundant phylum in the gut microbial communities of all bird species. Deferribacterota, Dadabacteria and Sumerlaeota were the most abundant in ACi. Samples from the same diet groups (i.e., CB and LA) tended to have similar gut microbial structures unrelated to the host phylogeny (Figure 3B).

A total of 1,005 genera were identified in the gut microbiome. The most dominant genera were *Clostridium* (Firmicutes phylum; 9.6% of sequences on average)*, Catellicoccu*s (Firmicutes; 5.7%), *Campylobacte*r (Proteobacteria; 5.4%), *Escherichia* (Proteobacteria; 2.5%), and *Helicobacter* (Epsilonbacteraeota; 2.7%). These genera were found in all samples. Among diet groups, *Catellicoccus* was the most dominant genus identified in the omnivorous (11.9%) and carnivorous (10.9%), and their abundance in the herbivorous was low (< 0.1%). *Clostridium* was the most dominant genus identified in the herbivorous (17.5%), their abundance in the gut of omnivorous (5.9%) and carnivorous birds (4.9%) were significantly lower (Supplementary Figure S3). Additionally, *Streptococcus* and *Campylobacter*, as frequently found species, were identified in avian guts.

A total of 3,871 ASVs for the herbivorous, 4,925 ASVs for the omnivorous, and 3,778 ASVs for the carnivorous were recovered (Figure 3C). Among these, 830 gut ASVs (13.5%, a total of 6,129 ASVs) shared among the three diet groups (Figure 3C). These shared ASVs mainly belonged to Proteobacteria (28.3%), Firmicutes (23.8%), and Bacteroidota (12.7%). The core ASVs were defined as those which occurred in over 50% of migratory birds in each diet group. The core ASVs were identified in the herbivorous (29 ASVs), omnivorous (34 ASVs), and carnivorous (8 ASVs), which differed among different diet groups (Supplementary Table S3). Only two core ASVs belonging to *Rhodococcus-erythropolis* (Actinobacteria) and *Ralastonia* (Proteobacteria) were shared by all diet groups (Figure 3D).

### Complexity and stability of gut microbial communities

**Figure 4:**
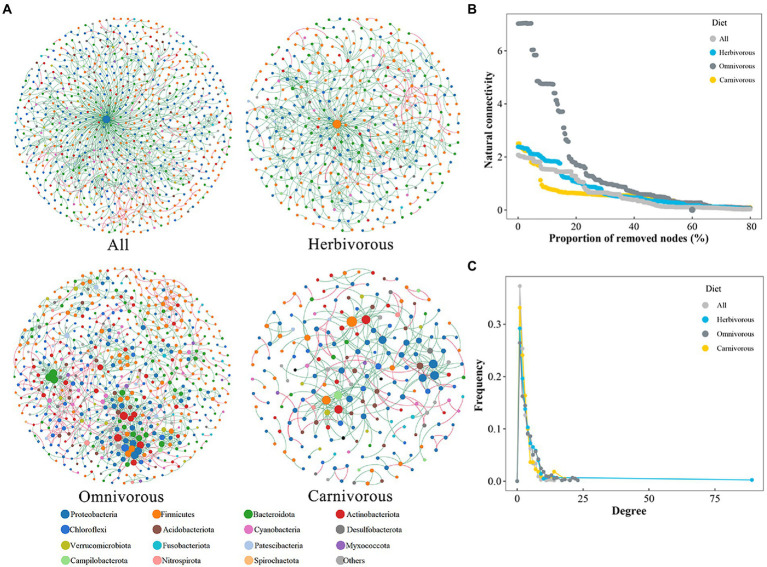
The phylogenetic molecular ecology networks and stability (pMENs) based on correlation analysis. **(A)** Networks among gut microbial ASVs. The network nodes are colored according to the taxonomy at the phylum level. A connection indicates a statistically significant (FDR-corrected *p*-value < 0.01) positive correlation (red) or a negative correlation (green). The size of each node is proportional to the relative abundance of the ASVs; the thickness of a connection between two nodes (i.e., an edge) is proportional to the value of Spearman’s correlation coefficient; **(B)** node degree distribution of the networks. **(C)** The robustness of gut microbiome network.

The pMENs of the gut microbiome at the ASV level among the different bird species were evaluated based on Pearson’s correlation coefficient (Figure 4A; Table 2). The overall topology indices showed that the distribution of all network connectivity fitted well with the power-law model (the range of *R*^2^ value was from 0.812 to 0.914). The omnivorous group had more edges than the carnivorous group in the networks. The average path length ranged from 4.629 to 9.392, demonstrating that the average network distance was variable among diet groups. The gut microbial community of the omnivores had the highest node connectivity, with an average degree of 4.169. Meanwhile, the node connectivity of the gut microbial community was the lowest in the omnivorous, with an average degree of 3.055. The value of the modularity index of the three groups was higher than 0.7, suggesting that each network had a modular structure. In pMENs, a higher proportion of negative associations were observed in the omnivorous than in the dietary specialization birds.

**Table 2:** The topological features of the gut microbial networks from the herbivorous, omnivorous and carnivorous.

Treatments	Average degree	Diameter	Modularity	Clustering coefficient	Average path length	Node	Edge	*R* square of power-law	Positive/negative
Herbivorous	3.547	4.07	0.764	0.177	4.791	428	759	0.812	51/708
Omnivorous	4.169	6.712	0.828	0.221	9.392	606	1,284	0.863	313/971
Carnivorous	3.055	3.739	0.761	0.176	4.529	220	336	0.914	76/260

Microbial networks for the herbivorous and carnivorous were less integrated than the omnivorous, with a lower average node degree (Figure 4B; Table 2) and greater decreased network stability upon node attack removal (Figure 4C; Table 2). The result showed that the gut microbiome of the omnivorous had the most complex associations, followed by the herbivorous and carnivorous.

### Relative importance of deterministic and stochastic processes

**Figure 5:**
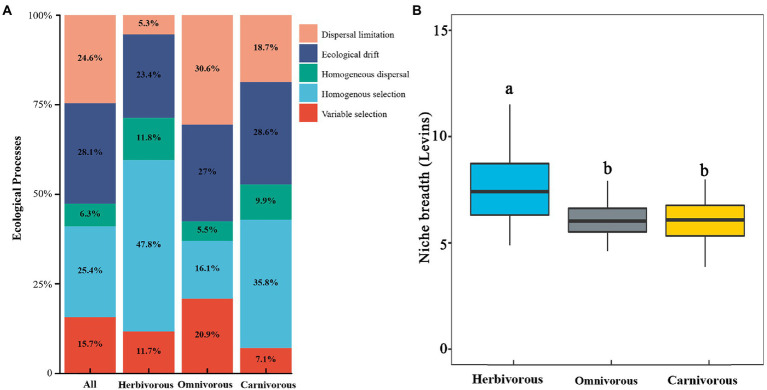
Ecological processes about the gut microbial community assembly. **(A)** Quantification of ecological processes governing the microbial community assembly and turnover for diets groups. The percentages are relative contributions of each process; **(B)** comparison of mean habitat niche breadth among herbivorous, carnivorous, and omnivorous birds. The different letters indicate a significant difference (*p* < 0.05 level using Tukey’s test).

Based on the metric of the weighted bacterial community assembly (βNTI) analysis, stochastic processes (58.9%) contributed more to the community composition of migratory birds than the deterministic process (41.1%, Figure 5A; Supplementary Figure S4A). For diet groups, the relative contribution of stochastic processes explained 63.1% of the gut microbiome assembly in the omnivorous, 57.1% of the gut microbiome assembly in the carnivorous, and 40.5% of the gut microbiome assembly in the herbivorous (Figure 5A; Supplementary Figure S4A). Homogeneous selection (relative contribution, 47.8% in herbivorous birds; 35.8% in carnivorous birds) was the major process driving the gut microbiome to convergence, while homogeneous dispersal (30.6%) was the major process driving omnivorous birds’ gut microbiome to convergence (Figure 5A; Supplementary Figure S4A). The drift contribution was much higher for the carnivorous (28.6%) than the herbivorous (5.2%, Figure 5A). The deterministic processes became more critical with increasing dietary specialization. Furthermore, gut microbiome exhibited significantly wider niche breadths in the herbivorous than in the carnivorous/ omnivorous (p <0.05; Figure 5B; Supplementary Figure S4B).

### Predictions of the function of the gut microbiome

**Figure 6:**
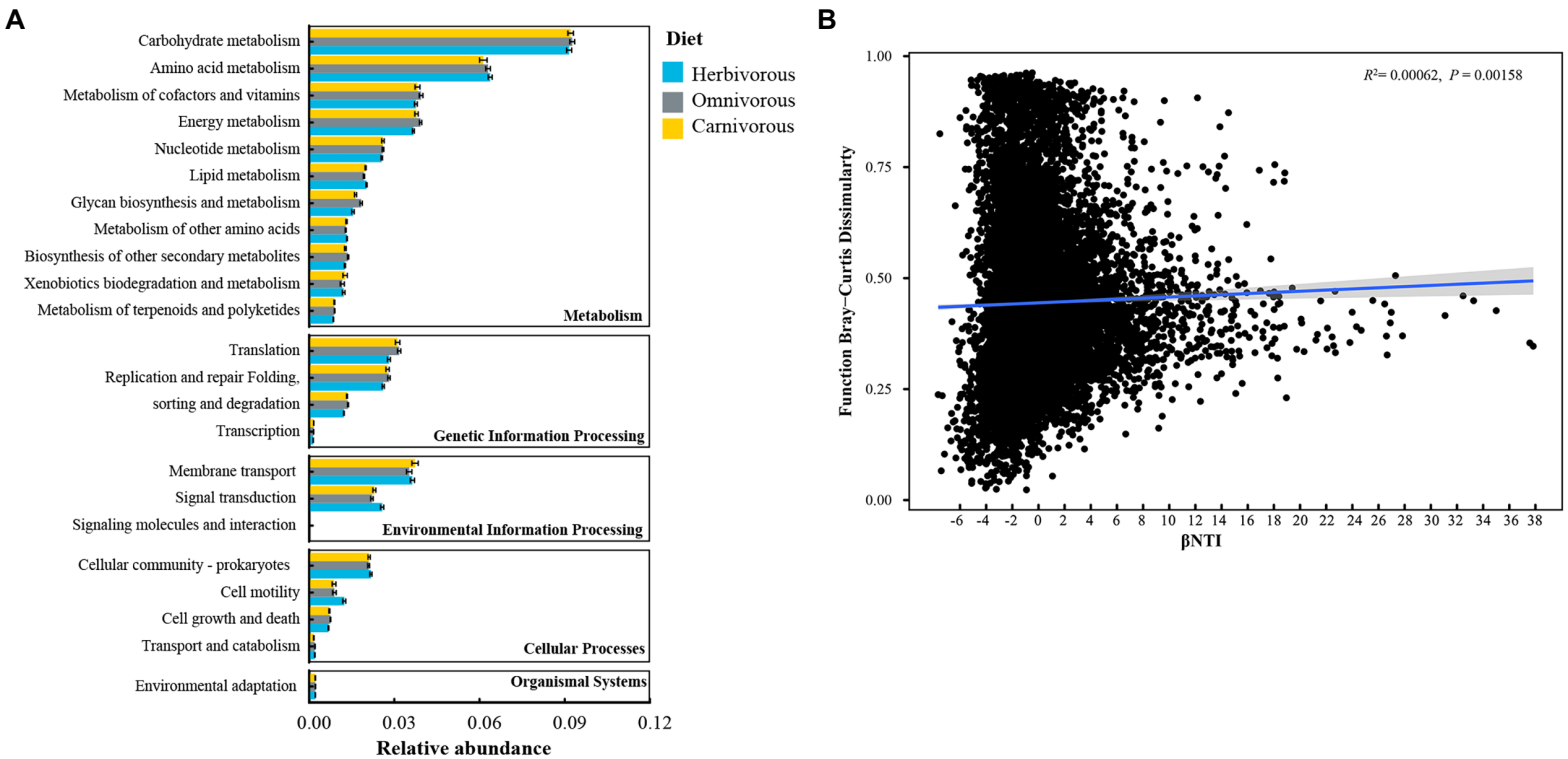
Predictions about the function during the microbial community assembly based on amplicon sequence variants (ASVs). **(A)** Level 2 KEGG orthologue functional predictions annotated by PICRUSt2; **(B)** the correction between βNTI and the function. Linear regression models (shown as blue lines) and associated correlation coefficients are provided on each panel.

Potential functions of all ASVs were annotated using the PICRUSt2. The majority of functions were clustered into metabolism (11 pathways), cellular processes (4 pathways), genetic information processing (4 pathways), environmental information processing (2 pathways), and organismal systems (1 pathway; Figure 6A). Among them, carbohydrate metabolism and amino acid metabolism were the two main metabolic pathways, and their relative abundance accounted for more than 5% of the whole process (Figure 6A). Most metabolic pathways were similar across the three diet groups (*p* > 0.05; Figure 6A).

The relationships between the β-nearest taxon index (βNTI) and functional Bray–Curtis dissimilarity were used to infer the impact of deterministic/stochastic assembly processes on the metabolic functions of the ASVs. The metabolic functions of the ASVs were significantly positively correlated with βNTI (Figure 6B).

## Discussion

### Diversification and function of gut microbiome among birds

Birds show a remarkable evolutionary diversification of strategies from dietary complex omnivorous mixtures as the ancestral trait to dietary specificity including herbivores and carnivores (Langen and Owens, 2002). In line with prior surveys of mammalian host species (Ley et al., 2008a,b; Perofsky et al., 2019), the omnivorous in this study exhibited the greatest gut microbial diversity, followed by the herbivorous and carnivorous (Figure 2). The diversity of the gut microbiota of migratory birds was correlated with a range of variations in food selection and diet could alter the gut microbial communities (Wu et al., 2011, 2018a,b). The microbial diversity and its versatility may allow the halobiont to function more optimally and adapt rapidly to changing conditions (Yachi and Loreau, 1999).

The predicted function of the gut microbiome reflected differences in carbohydrate and amino acid utilization associated with diet groups of migratory birds (Figure 6A). As found for the herbivorous, the gut microbiome was enriched for folivorous pathways associated with increased plant fiber degradation, such as metabolism of cofactors and vitamins, and lipid metabolism (Moschen et al., 2012; Yang et al., 2016). Along with the carnivorous (Perofsky et al., 2019), the omnivorous microbiome was also enriched in pathways related to energy metabolism, translation, and glycan biosynthesis and metabolism. However, there have been insufficient dietary studies of the omnivorous to compare the differences in plant and animal consumption. In the omnivorous, most of the identified core ASVs, i.e., *Lactobacillus, Bacteroides plebeius,* and *Hydrogenophaga,* in our study were reported to perform essential functions in nutrition uptake, health promotion, and pathogen defense for their hosts (Thomas et al., 2011; Grond et al., 2019). In all diet groups, the shared core ASV, i.e., *Rhodococcus-erythropolis*, can produce enzymes that allow them to carry out an enormous number of bioconversions and degradations (Carvalho and Fonseca, 2005). Despite highly divergent gut microbiota compositions among dietary groups (Figure 2A), predicted community functional traits were quite similar among the different dietary groups (Figure 6A), indicating a high degree of functional redundancy among migratory birds’ gut microbiome (Lozupone et al., 2012). It is noted that our conclusions concerning diet-associated signals in gut metagenomes of migratory birds are predictive, future research on the function of the gut microbiome is required to obtain a clear picture.

### Diet as the dominant factor contributing to variation in the gut microbiome

This study is an initial effort to understand the gut microbial community of migratory birds in the PYL wetland. Our results revealed that the gut microbiome was similar among the same diet groups (Table 2; Figure 2), and both diet and host phylogeny play important roles in the gut microbial community assembly of migratory birds. The effect value of diet combined with host phylogeny on the difference in gut microbiome was 30.0%, and diet (20.1%) was identified as the dominant factor. Diets for migratory birds are a compound factor. Migratory birds change diets during migration, which makes them subjected to different local microorganisms associated with food sources. Local diet as a potential driver of gut microbiome assembly has been shown in a study of the gut microbiota of songbirds, which identified similar gut microbial communities within and among species during the stopovers of the songbirds along the migratory route (Lewis et al., 2017). Different diets also require distinct digestive mechanisms and may need specialized microbial communities in the gut to assist in effective digestion (Grond et al., 2018). Microbes originating from diets are the major sources of microbial colonizers for gut microbes. Selective filtering at the colonization stage may be controlled by the host or microbe characteristics and stochastic processes (Bisson et al., 2009; Sieber et al., 2019).

### Effects of dietary specialization on the gut microbial communities

Network analysis provided another view of microbial interactions and ecological rules for community assembly (Grond et al., 2019; Song et al., 2020). Although higher in similarity threshold, the networks of the gut microbiome in dietary specialized (herbivorous and carnivorous) birds showed lower numbers of nodes, edges, and lower average connectivity (*p* < 0.05) than those of the omnivorous (Table 2). This indicates fewer interactions in dietary specialized birds than those in the omnivorous, which implies that biodiversity plays a role in the ecosystem stability in microbial communities (Feng et al., 2017). These results implicate that dietary specialization drove gut microbial communities toward a single direction, resulting in relatively less diverse gut microbiota (Ge et al., 2021). Besides, the gut microbiome of the herbivorous occupied a wide variety of niches (i.e., utilized an array of resources; Figure 4B), which was expected to be metabolically more flexible at the community level than one with a narrow niche breadth (Wu et al., 2018a,b; Jiao et al., 2020). Microbiomes with very different taxonomic compositions could share a substantial functional similarity, which is generally believed to play a role in the stability of microbial functions during disturbances. The functional redundancy in the microbial community may provide an environmental health advantage (Le Chatelier et al., 2013; Blakeley-Ruiz et al., 2019). Therefore, the herbivorous can adapt to changes in food sources.

Dietary specialization significantly altered the assembly processes that shape the gut microbiome of migratory birds. For example, the dispersal limitation decreased, and homogeneous selection increased in the dietary specialization birds (Figure 4A). This result suggests that the dispersal limitation leads to a relatively decentralized accumulation of gut microbial diversity in migratory birds with different diets. Specialized diets narrowed the range of food and foraging environments (Wiens, 2011; Shafquat et al., 2014; Burns et al., 2015), which limits the dispersal of microorganisms (Ley et al., 2008a,b; Muegge et al., 2011). Furthermore, migratory birds formed different ecological niches, which reduced the contact among birds (Ge et al., 2021). Additionally, the homogeneous selection may reflect the choice of the gut microbiome in the same inner intestinal tract environment, which maintained the similarity in different species of the same diet (Philipp and Moran, 2013). Dietary specialization had a larger βNTI value than the omnivorous (Figure 5A; Supplementary Figure S4A; Figure 6B). We speculate that after a long period, when the cumulative historical stochasticity causes a substantial change of host genetic identity, the host genetic isolation occurs and results in different host-bacterium specificity (Ge et al., 2021). The specialized function of gut microbes could lead to less available nutrients and more competition for microorganisms. But in birds, each organism specializes in different diets, and there would be less competition.

## Conclusion

This study evaluated the contributions of diet and host phylogeny on the assembly of the gut microbiome of migratory birds. Diet was identified as the dominant factor contributing to observed variations of gut microbial structures among migratory birds. Omnivorous species had significantly higher gut microbiome diversity than herbivorous and carnivorous species. Dietary specialization also affected the balance between the deterministic and stochastic assembly of the gut microbiome. Stochastic processes were decreased as dietary specialization increased, indicating an increasing influence of selective processes. These insights could advance our understanding of host-microbiota interactions and the evolution of migratory birds’ dietary flexibility and diversification.

## Data availability statement

The datasets presented in this study can be found in online repositories. The names of the repository/repositories and accession number(s) can be found at: https://www.ncbi.nlm.nih.gov/, PRJNA736842.

## Author contributions

BW: conceptualization, methodology, data curation, formal analysis, visualization, and writing—original draft. HZ: data curation, formal analysis, and visualization. YL: investigation and formal analysis. LR: resources and revision. ZK: investigation, revision, and formal analysis. XM: revision and supervision. LW: writing—review and editing, funding acquisition, and supervision. All authors contributed to the article and approved the submitted version.

## Funding

This work was supported by the National Natural Science Foundations of China (grant no. 31971470), the key science grant of Jiangxi Province (20212ACB205009), the National Key R&D Program of China (2019YFC0605005), and the Youth Science Fund Project in Jiangxi Province (2018BA214004).

## Conflict of interest

The authors declare that the research was conducted in the absence of any commercial or financial relationships that could be construed as a potential conflict of interest.

## Publisher’s note

All claims expressed in this article are solely those of the authors and do not necessarily represent those of their affiliated organizations, or those of the publisher, the editors and the reviewers. Any product that may be evaluated in this article, or claim that may be made by its manufacturer, is not guaranteed or endorsed by the publisher.

## Footnotes

^1^ http://blast.ncbi.nlm.nih.gov/Blast.cgi

^2^ https://www.megasoftware.net

^3^ http://www.qiime2.org

^4^ https://img.jgi.doe.gov/cgi-bin/m/main.cgi/m/main.cgi

## Supplementary material

SUPPLEMENTARY FIGURE S1 Rarefaction curves of the gut microbiome.

SUPPLEMENTARY FIGURE S2 Comparison of gut microbial diversity of 17 species of birds. The index of Shannon, Simpson, Chao 1, and Faith's phylogenetic diversity for each species. Differences in diversity were evaluated using the Kruskal test adjusted for multiple comparisons.

SUPPLEMENTARY FIGURE S3 Phylum: Pie charts of microbial composition for the abundant phyla (> 1%). Genus: Pie charts of microbial composition for the abundant genera (> 1%).

SUPPLEMENTARY FIGURE S4 **(A)** Quantification of ecological processes governing the microbial community assembly and turnover in the gut microbial communities. **(B)** Comparison of average habitat niche breadth of gut microbial species.
