# Supplementary figures and images for: Diet drives the gut microbiome composition and assembly processes in winter migratory birds in the Poyang Lake wetland, China
Source: Front Microbiol. 2022 Sep 23;13:973469. doi: 10.3389/fmicb.2022.973469 (PMC9537367; doi:10.3389/fmicb.2022.973469)

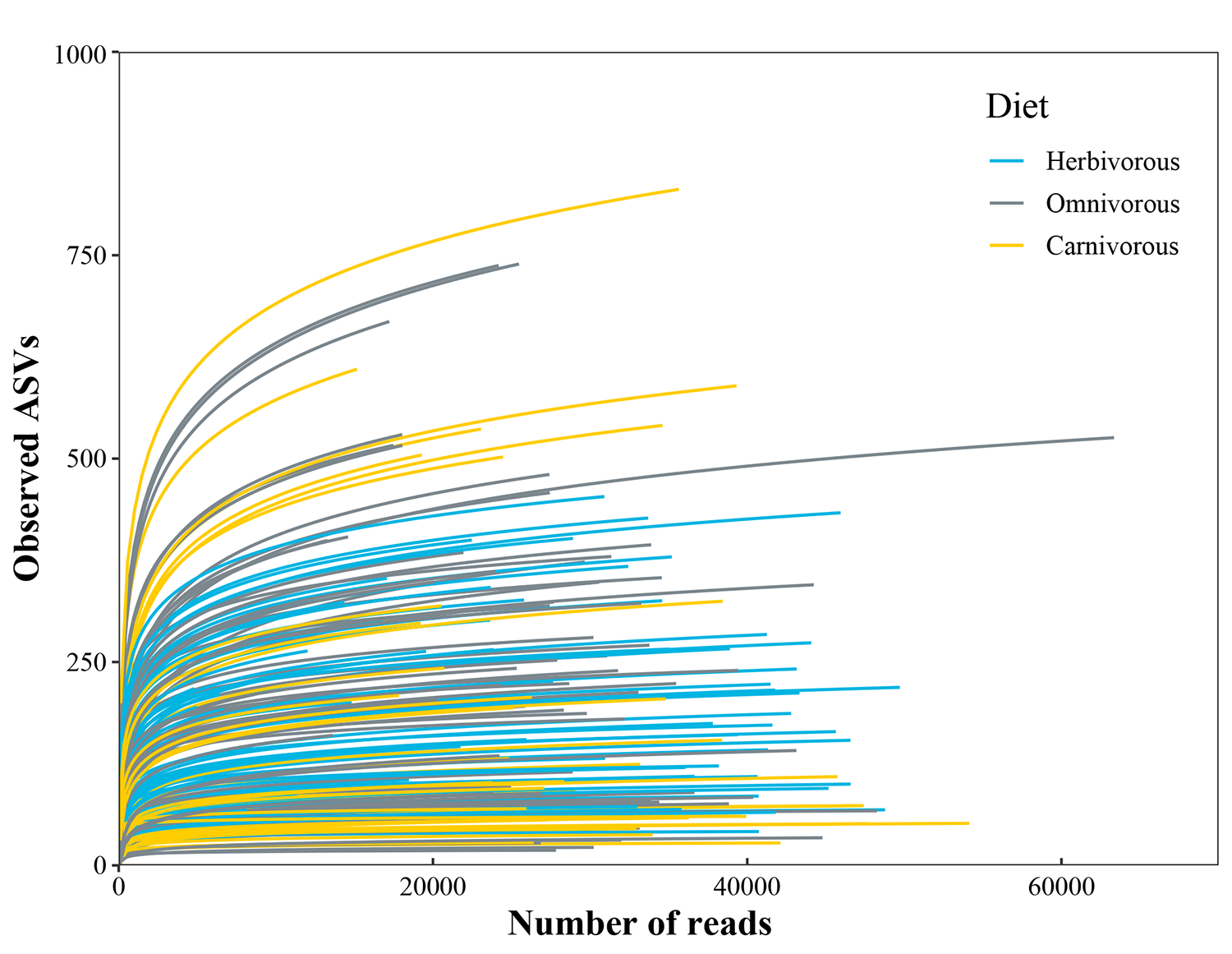

Supplement: SUPPLEMENTARY FIGURE S1 — Rarefaction curves of the gut microbiome. [file Image_1.TIF]

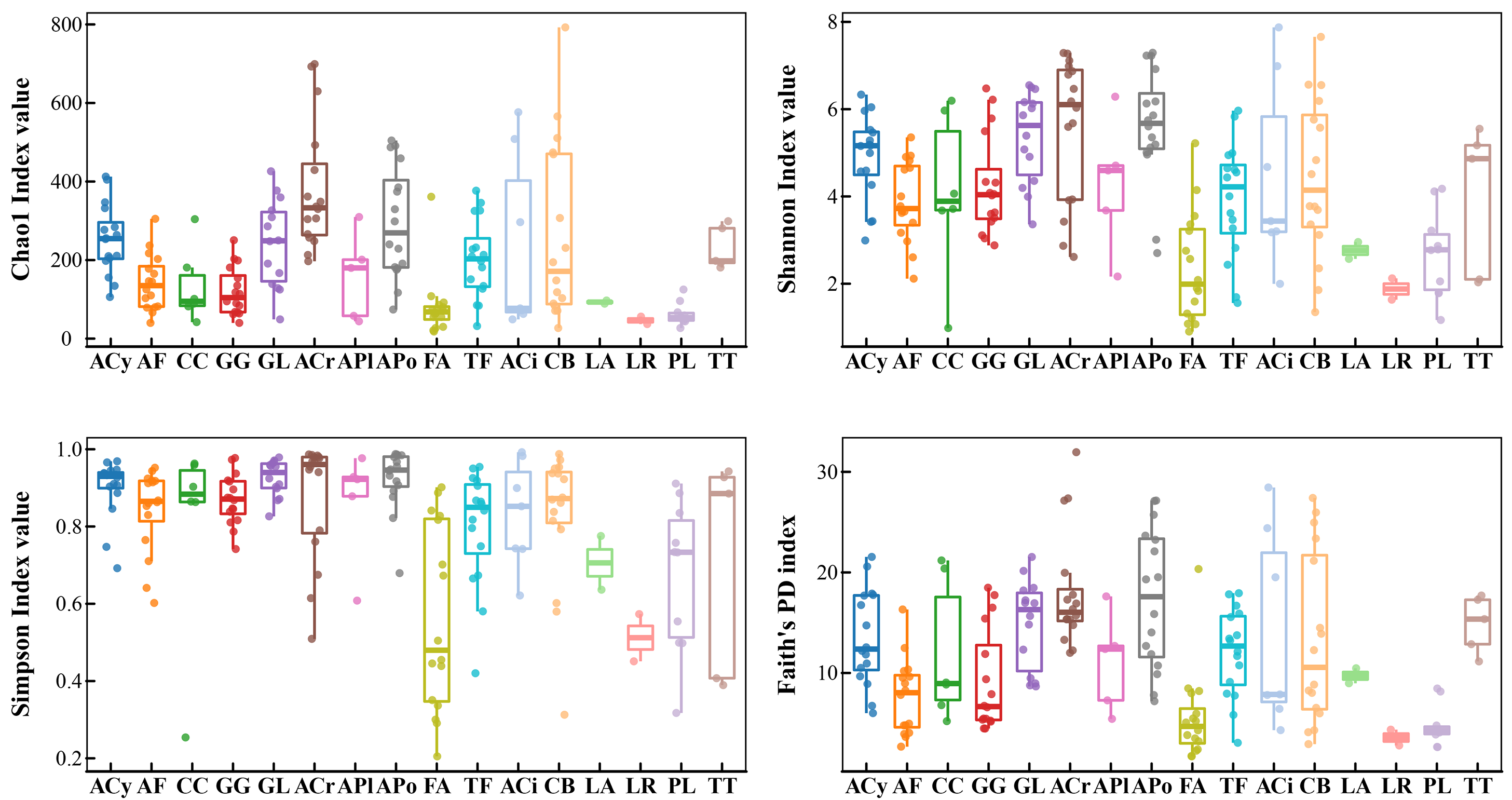

Supplement: SUPPLEMENTARY FIGURE S2 — Comparison of gut microbial diversity of 17 species of birds. The index of Shannon, Simpson, Chao 1, and Faith's phylogenetic diversity for each species. Differences in diversity were evaluated using the Kruskal test adjusted for multiple comparisons. [file Image_2.TIF]

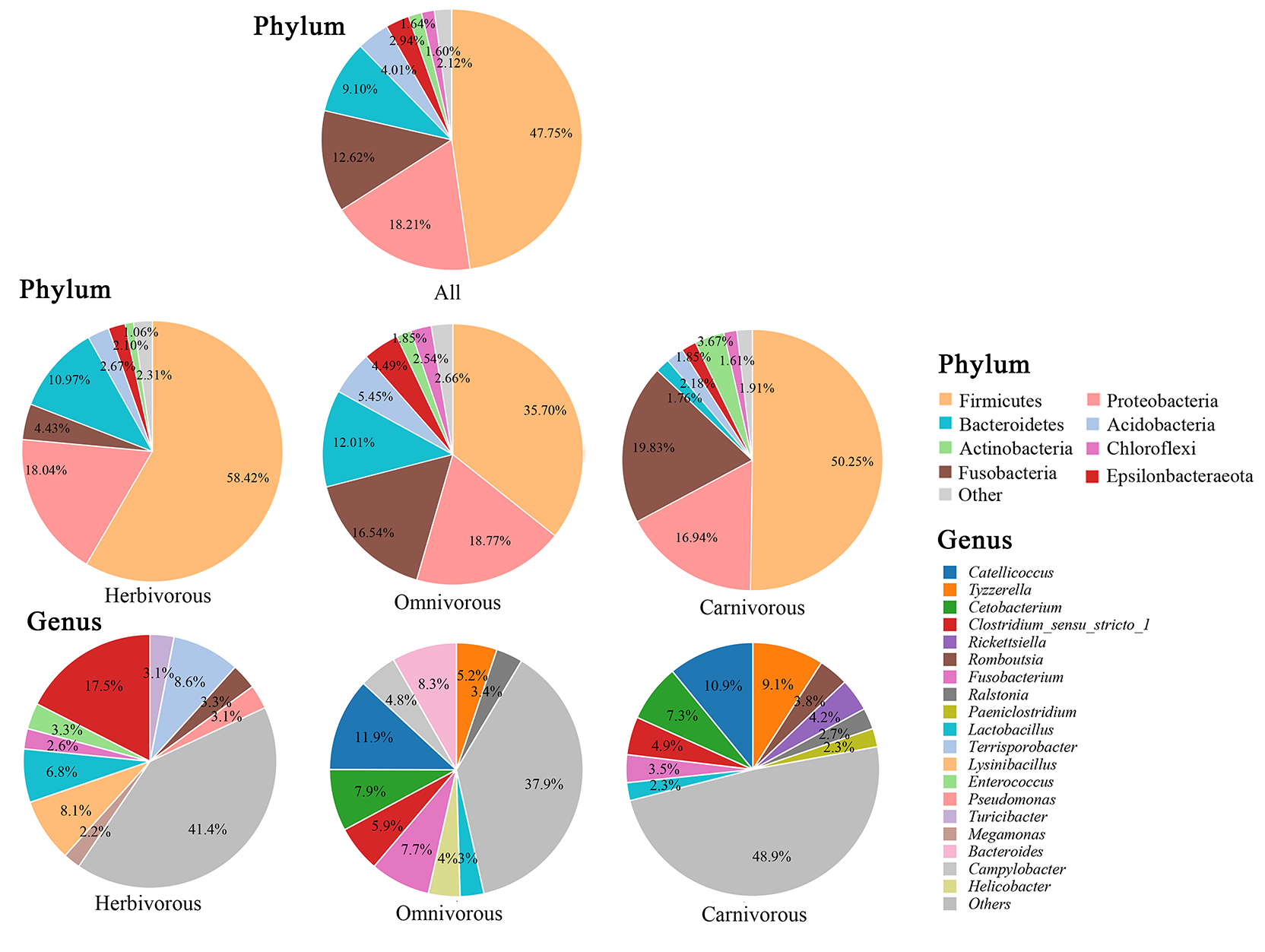

Supplement: SUPPLEMENTARY FIGURE S3 — Phylum: Pie charts of microbial composition for the abundant phyla (> 1%). Genus: Pie charts of microbial composition for the abundant genera (> 1%). [file Image_3.TIF]

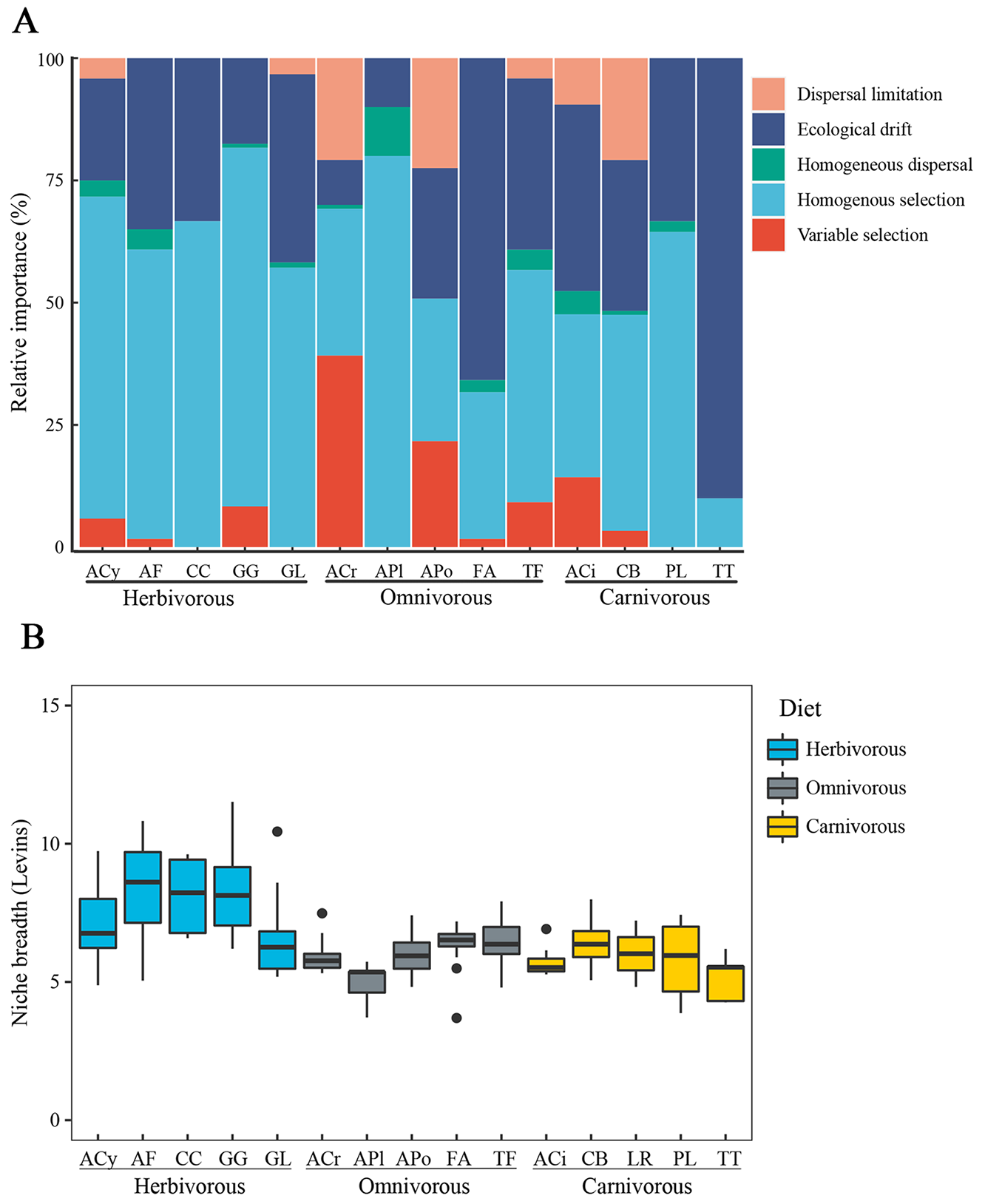

Supplement: SUPPLEMENTARY FIGURE S4 — (A) Quantification of ecological processes governing the microbial community assembly and turnover in the gut microbial communities. (B) Comparison of average habitat niche breadth of gut microbial species. [file Image_4.TIF]
