# Supplementary material for: Diet drives the gut microbiome composition and assembly processes in winter migratory birds in the Poyang Lake wetland, China
Source: Front Microbiol. 2022 Sep 23;13:973469. doi: 10.3389/fmicb.2022.973469 (PMC9537367; doi:10.3389/fmicb.2022.973469)
Supplement: Supplementary file 5 [file Table_1.docx]

**Supplemental Tables**

**Table S1** The alpha diversity of gut microbiome among host bird species

| Bird species | Chao1 | Faith's PD | Shannon | simpson | Good's coverage |
| --- | --- | --- | --- | --- | --- |
| ACi | 216.13 ± 82.469 | 14.113 ± 3.697 | 4.478 ± 0.823 | 0.831 ± 0.052 | 0.955 ± 0.018 |
| ACr | 312.361 ± 33.926 | 18.05 ± 1.447 | 5.564 ± 0.413 | 0.873 ± 0.039 | 0.936 ± 0.009 |
| ACy | 201.032 ± 19.632 | 13.521 ± 1.191 | 4.899 ± 0.243 | 0.901 ± 0.019 | 0.961 ± 0.004 |
| AF | 120.291 ± 15.428 | 7.65 ± 0.942 | 3.892 ± 0.231 | 0.838 ± 0.028 | 0.979 ± 0.003 |
| APL | 146.712 ± 58.484 | 11.081 ± 2.16 | 4.287 ± 0.676 | 0.863 ± 0.064 | 0.975 ± 0.01 |
| APo | 261.587 ± 31.922 | 17.368 ± 1.739 | 5.595 ± 0.335 | 0.921 ± 0.02 | 0.951 ± 0.007 |
| CB | 231.286 ± 50.964 | 13.282 ± 2.147 | 4.432 ± 0.459 | 0.827 ± 0.045 | 0.953 ± 0.011 |
| CC | 131.708 ± 37.957 | 11.915 ± 2.868 | 4.101 ± 0.773 | 0.8 ± 0.111 | 0.977 ± 0.008 |
| FA | 68.218 ± 19.058 | 5.605 ± 1.127 | 2.363 ± 0.314 | 0.555 ± 0.061 | 0.988 ± 0.004 |
| GG | 118.962 ± 16.152 | 9.142 ± 1.278 | 4.249 ± 0.285 | 0.871 ± 0.016 | 0.982 ± 0.003 |
| GL | 207.998 ± 26.969 | 14.871 ± 1.189 | 5.355 ± 0.281 | 0.926 ± 0.012 | 0.964 ± 0.006 |
| LA | 79.363 ± 7.748 | 9.723 ± 0.746 | 2.762 ± 0.193 | 0.688 ± 0.079 | 0.985 ± 0.001 |
| LR | 34.125 ± 5.125 | 3.579 ± 0.785 | 1.879 ± 0.245 | 0.504 ± 0.075 | 0.994 ± 0.001 |
| PL | 45.792 ± 7.325 | 4.744 ± 0.634 | 2.673 ± 0.315 | 0.67 ± 0.063 | 0.993 ± 0.002 |
| TF | 170.488 ± 22.607 | 12.036 ± 1.096 | 3.928 ± 0.332 | 0.797 ± 0.037 | 0.967 ± 0.004 |
| TT | 211.956 ± 30.106 | 14.87 ± 1.263 | 3.944 ± 0.774 | 0.702 ± 0.131 | 0.957 ± 0.006 |
