# Supplementary material for: Diet drives the gut microbiome composition and assembly processes in winter migratory birds in the Poyang Lake wetland, China
Source: Front Microbiol. 2022 Sep 23;13:973469. doi: 10.3389/fmicb.2022.973469 (PMC9537367; doi:10.3389/fmicb.2022.973469)
Supplement: Supplementary file 6 [file Table_2.docx]

**Supplemental Tables**

**Table S2** ANOSIM and ADONIS of gut microbiome based on Bray-Curtis and Weighted Unifrac dissimilarity among different diet groups

|  |  | Bray-Curits | |  |  | Weighted Unifrac |  |  |
| --- | --- | --- | --- | --- | --- | --- | --- | --- |
|  |  | ANOSIM |  | ADONIS |  | ANOSIM | ADONIS |  |
|  |  | R | *P* | R^2^ | *P* | R *P* | R^2^ | *P* |
| Carnivorous/Omnivorous | | 0.1402 | 0.001 | 0.0313 | 0.001 | -0.0084 0.599 | 0.03174 | 0.004 |
| Carnivorous/Herbivorous | | 0.664 | 0.001 | 0.0927 | 0.001 | 0.1586 0.001 | 0.0656 | 0.001 |
| Omnivorous/Herbivorous | | 0.3506 | 0.001 | 0.0759 | 0.001 | 0.1161 0.0015 | 0.528 | 0.001 |
