# Supplementary material for: Diet drives the gut microbiome composition and assembly processes in winter migratory birds in the Poyang Lake wetland, China
Source: Front Microbiol. 2022 Sep 23;13:973469. doi: 10.3389/fmicb.2022.973469 (PMC9537367; doi:10.3389/fmicb.2022.973469)
Supplement: Supplementary file 7 [file Table_3.docx]

**Supplemental Tables**

Table S3 The core ASVs identified of the bird samples in diet groups.

| Groups | Phylum |  | Class |  | Order |  | Family |  | Genus | 1 |
| --- | --- | --- | --- | --- | --- | --- | --- | --- | --- | --- |
| Herbivorous | Firmicutes | 22 | Bacilli | 7 | Erysipelotrichales | 1 | Erysipelotrichaceae | 1 | *Turicibacter* | 1 |
|  |  |  |  |  | Lactobacillales | 1 | Lactobacillaceae | 1 | *Lactobacillus* | 1 |
|  |  |  |  |  | Bacillales | 1 | Planococcaceae | 1 | *Psychrobacillus* | 1 |
|  |  |  |  |  | Paenibacillales | 1 | Paenibacillaceae | 1 | *Paenibacillus* | 1 |
|  |  |  |  |  | Bacillales | 1 | Planococcaceae | 1 | *Lysinibacillus* | 1 |
|  |  |  |  |  | Paenibacillales | 1 | Paenibacillaceae | 1 | *Paenibacillus* | 1 |
|  |  |  |  |  | Bacillales | 1 | Planococcaceae | 1 | *Lysinibacillus* | 1 |
|  |  |  | Clostridia | 15 | Peptostreptococcales-Tissierellales | 1 | Peptostreptococcaceae | 1 | *Terrisporobacter* | 1 |
|  |  |  |  |  | Clostridiales | 9 | Clostridiaceae | 9 | *Clostridium_sensu_stricto_1* | 9 |
|  |  |  |  |  | Lachnospirales | 1 | Lachnospiraceae | 1 | *Cellulosilyticum* | 1 |
|  |  |  |  |  | Peptostreptococcales-Tissierellales | 1 | Peptostreptococcaceae | 1 | *Romboutsia* | 1 |
|  |  |  |  |  | Lachnospirales | 1 | Lachnospiraceae | 1 | *Cellulosilyticum* | 1 |
|  |  |  |  |  | Peptostreptococcales-Tissierellales | 1 | Peptostreptococcaceae | 1 | *Romboutsia* | 1 |
|  | Proteobacteria | 3 | Alphaproteobacteria | 2 | Rhizobiales | 1 | Xanthobacteraceae | 1 |  |  |
|  |  |  |  |  | Rhizobiales | 1 | Beijerinckiaceae | 1 | *Methylobacterium-Methylorubrum* | 1 |
|  |  |  | Gammaproteobacteria |  | Burkholderiales | 1 | Burkholderiaceae | 1 | *Ralstonia* | 1 |
|  | Campilobacterota | 2 | Campylobacteria | 2 | Campylobacterales | 2 | Helicobacteraceae | 1 | *Helicobacter* | 1 |
|  |  |  |  |  |  | 1 | Campylobacteraceae | 1 | *Campylobacter* | 1 |
|  | Actinobacteriota | 1 | Actinobacteria | 1 | Corynebacteriales | 1 | Nocardiaceae | 1 | *Rhodococcus* | 1 |
|  | Fusobacteriota | 1 | Fusobacteriia | 1 | Fusobacteriales | 1 | Fusobacteriaceae | 1 | *Fusobacterium* | 1 |
| Omnivorous | Firmicutes | 14 | Clostridia | 11 | Oscillospirales | 6 | Oscillospiraceae | 2 | *UCG-005* | 1 |
|  |  |  |  |  |  |  |  |  | *Colidextribacter* | 1 |
|  |  |  |  |  |  |  | Ruminococcaceae | 4 | uncultured | 1 |
|  |  |  |  |  |  |  |  |  | *Faecalibacterium* | 1 |
|  |  |  |  |  |  |  |  |  | *Subdoligranulum* | 2 |
|  |  |  |  |  | Lachnospirales | 1 | Lachnospiraceae | 1 | *Tyzzerella* | 1 |
|  |  |  |  |  | Clostridiales | 1 | Clostridiaceae | 1 | *Clostridium_sensu_stricto_1* | 1 |
|  |  |  |  |  | Clostridia |  | Oscillospirales | 1 | *Colidextribacter* | 1 |
|  |  |  |  |  | Peptostreptococcales-Tissierellales | 2 | Peptostreptococcaceae | 2 | *Romboutsia* | 1 |
|  |  |  |  |  |  |  |  |  | *Terrisporobacter* | 1 |
|  |  |  | Bacteria |  | Lactobacillales |  | Streptococcaceae |  | *Streptococcus* | 1 |
|  |  |  | Bacilli | 2 | Erysipelotrichales |  | Erysipelotrichaceae |  | *Turicibacter* | 1 |
|  |  |  |  |  | Lactobacillales |  | Lactobacillaceae |  | *Lactobacillus* | 1 |
|  | Proteobacteria | 11 | Alphaproteobacteria | 8 | Rhodobacterales | 1 | Rhodobacteraceae | 1 |  |  |
|  |  |  |  |  | Rhizobiales |  | Beijerinckiaceae |  | *Methylocystis* | 1 |
|  |  |  |  |  | Rhizobiales | 3 | Xanthobacteraceae | 1 | *Bradyrhizobium* | 1 |
|  |  |  |  |  |  |  | Beijerinckiaceae | 1 | *Methylobacterium-Methylorubrum* | 1 |
|  |  |  |  |  |  |  | Rhizobiales_Incertae_Sedis | 1 | uncultured | 1 |
|  |  |  |  |  | Sphingomonadales |  | Sphingomonadaceae |  | *Sphingorhabdus* | 1 |
|  |  |  |  |  | Rhizobiales | 2 | Beijerinckiaceae | 2 | *Bosea* | 1 |
|  |  |  |  |  |  |  |  |  | *alphaI_cluster* | 1 |
|  |  |  | Gammaproteobacteria | 3 | Burkholderiales | 2 | Comamonadaceae | 1 | *Hydrogenophaga* | 1 |
|  |  |  |  |  |  |  | Burkholderiaceae | 1 | *Ralstonia* | 1 |
|  |  |  |  |  | Enterobacterales |  | Enterobacteriaceae | 1 | *Escherichia-Shigella* | 1 |
|  | Bacteroidota | 5 | Bacteroidia | 5 | Bacteroidales | 5 | Bacteroidaceae | 4 | *Bacteroides* | 4 |
|  |  |  |  |  |  |  | Prevotellaceae | 1 | uncultured | 1 |
|  | Fusobacteriota | 2 | Fusobacteriia | 2 | Fusobacteriales | 2 | Fusobacteriaceae | 2 | *Fusobacterium* | 2 |
|  | Actinobacteriota | 1 | Actinobacteria | 1 | Corynebacteriales | 1 | Nocardiaceae | 1 | *Rhodococcus* | 1 |
|  | Campilobacterota | 1 | Campylobacteria | 1 | Campylobacterales | 1 | Helicobacteraceae | 1 | *Helicobacter* | 1 |
| Carnivorous | Firmicutes | 5 | Clostridia | 5 | Peptostreptococcales-Tissierellales | 2 | Peptostreptococcaceae | 2 | *Paeniclostridium* | 1 |
|  |  |  |  |  |  |  |  |  | *Paraclostridium* | 1 |
|  |  |  |  |  | Lachnospirales | 1 | Lachnospiraceae | 1 | *Tyzzerella* | 1 |
|  |  |  |  |  | Clostridiales | 1 | Clostridiaceae | 1 | *Clostridium_sensu_stricto_1* | 1 |
|  |  |  |  |  | Oscillospirales | 1 | Butyricicoccaceae | 1 | *Butyricicoccus* | 1 |
|  | Fusobacteriota |  | Fusobacteriia |  | Fusobacteriales | 1 | Fusobacteriaceae | 1 | *Fusobacterium* | 1 |
|  | Actinobacteriota |  | Actinobacteria |  | Corynebacteriales | 1 | Nocardiaceae | 1 | *Rhodococcus* | 1 |
|  | Proteobacteria |  | Gammaproteobacteria |  | Burkholderiales | 1 | Burkholderiaceae | 1 | *Ralstonia* | 1 |
